# Carbohydrate-binding domain CBM63 of microbial expansin-like *Bs*EXLX1 facilitates the adsorption of expansin-related proteins to hemicelluloses in plant secondary cell walls

**DOI:** 10.1186/s13068-025-02674-x

**Published:** 2025-07-09

**Authors:** Pramod Sivan, Deepika Dahiya, Ylenia Jabalera, Taru Koitto, Raul Perez-Jimenez, Ewa J. Mellerowicz, Emma Master, Francisco Vilaplana

**Affiliations:** 1 Division of Glycoscience, Department of Chemistry, KTH Royal Institute of Technology, AlbaNova University Centre, 106 91 Stockholm, Sweden; 2 Department of Bioproducts and Biosystems, School of Chemical Engineering, Aalto University, 02150 Espoo, Finland; 3 CIC bioGUNE BRTA, Bizkaia Science and Technology Park 801A, 48160 Derior, Spain; 4 Umeå Plant Science Centre, Department of Forest Genetics and Plant Physiology, Swedish University of Agricultural Sciences, 901 83 Umeå, Sweden; 5 Wallenberg Wood Science Centre (WWSC), KTH Royal Institute of Technology, Teknikringen 56-58, 100 44 Stockholm, Sweden; 6 Department of Chemical Engineering and Applied Chemistry, University of Toronto, 200 College Street, Toronto, ON M5S 3E5 Canada

**Keywords:** Expansin, Loosenin, Secondary cell wall, Hemicelluloses

## Abstract

**Background:**

Overcoming lignocellulose recalcitrance to enzymatic or chemical processing is a prerequisite for biorefinery applications. Expansins and loosenins are non-lytic proteins that could assist reducing this recalcitrance by disrupting the intermolecular contacts between plant cell wall components. Here, immunolocalization with fluorescence and transmission electron microscopy (TEM) were used to study the ability of a *Bacillus subtilis* expansin-like protein (*Bs*EXLX1), a *Phanerochaete carnosa* loosenin protein (*Pca*LOOL12) and a fusion protein of *Pca*LOOL12 with the carbohydrate-binding module 63 (CBM63) of *Bs*EXLX1 (i.e., *Pca*LOOL12-CBM63) to bind secondary cell walls (SCW) of aspen fibres, including fresh aspen wood, milled wood fibres (MWF) and MWF subjected to subcritical water extraction.

**Results:**

The immunofluorescence labelling of fresh wood samples showed a weak signal for *Pca*LOOL12 and a strong signal for *Bs*EXLX1 and *Pca*LOOL12-CBM63, suggesting the importance of CBM63 for protein adsorption to SCW components. TEM analysis after immunogold labelling revealed the presence of *Bs*EXLX1 and *Pca*LOOL12-CBM63 in all secondary cell wall layers. Pretreatment of wood samples with the proteins reduced the binding of glucomannan- and glucuronoxylan-specific monoclonal antibodies. Similarly, protein adsorption to MWF was higher before subcritical water extraction. Together, these results suggest the adsorption of *Bs*EXLX1 and *Pca*LOOL12-CBM63 to SCWs was mediated at least in part by their interaction with hemicelluloses.

**Conclusions:**

Our study demonstrates that microbial expansin-related proteins can bind to the secondary walls of aspen wood through potential interaction of CBM63 with hemicelluloses.

## Introduction

Cell growth in plants involves changes in the structural organization of the polymeric network of cellulose and matrix polysaccharides in primary cell walls [1, 2]. The family of α-expansin (EXPA) proteins are involved in acid-induced cell wall loosening and are responsible for structural changes in the primary cell wall crucial for wood formation [3, 4]. The related β-expansin protein family (EXPB), which include grass pollen allergens, are known to facilitate intracellular pollen tube invasion, whereas two other plant expansin subfamilies, expansin-like A (EXLA) and expansin-like B (EXLB) have not been so far functionally analysed [5]. Xylem cell growth is followed by the deposition of a thick multilayered secondary wall (SCW), characterized by aligned cellulose microfibrils embedded in a complex matrix of glucuronoxylan and glucomannan hemicelluloses, which become impregnated with polyphenolic lignin together with surrounding primary cell walls. This complex structure and chemistry of wood cell walls contribute to the highly recalcitrant properties of lignocellulose biomass against its degradation. 

Microbial enzymes from wood-degrading microorganisms are well known for their ability to disassemble cell walls. They include glycoside hydrolases (cellulases, xylanases, mannanases, pectinases), polysaccharide lyases (pectate lyases, rhamnogalacturonan lyases), oxidoreductases (lytic polysaccharide monooxygenases, LPMOs), and ligninases (laccases, peroxidases, polyphenol oxidases) that can disintegrate the cell wall causing the decomposition of lignocellulosic biomass [6]. These enzymes are widely used for commercial applications requiring saccharification of complex cell wall polymers into monomers [7]. However, the potential application of lignocellulosic biomass components into functional materials require their modification without complete disassembly. In this regard, microbial expansin-related proteins appear as promising biotechnological tools to disrupt the assembly of cellulosic fibres through biophysical effects, leading to an increased accessibility of catalytic enzymes to the glycan substrates during the biochemical conversion of biomass [8–10].

Microbial expansin-like proteins (EXLXs) have been identified in plant pathogens and saprotrophs suggesting their role in plant biomass decomposition [8]. Structurally, EXLXs comprise an N-terminal six-stranded double-Ѱ beta-barrel (DPBB) domain (D1) homologous to GH45 glycoside hydrolase family (GH) and a C-terminal domain (D2) that belongs to type-A CBM family 63 [11]. Other expansin-related proteins can have additional domains, such as a family 2 carbohydrate-binding module (CBM2), family 5 glycoside hydrolase (GH5) domain [12, 13], or GH9 domain [14]. Similarly, swollenins retain the core EXLX structure but have an N-terminal CBM1 (a type-A CBM) and fibronectin III insertion [15]. By contrast, microbial expansin-related proteins that lack the C-terminal CBM63 are classified as loosenins and ceratoplatanins [16–18].

The biophysical mechanisms responsible for the relaxation of plant cell walls by expansins involve the disruption of noncovalent interactions between cellulose microfibrils and hemicelluloses, or between tight junctions between neighbouring microfibrils, leading to cellulose microfibril slippage causing cell wall creep [19, 20]. The ability of *Bacillus subtilis* expansin *BsEXLX1* to induce extension of alkali-pretreated wheat coleoptiles and weaken filter paper was demonstrated, and subsequent mutations in the CBM63 domain demonstrated this domain was necessary for creep through selective interaction of the CBM63 with cellulose microfibrils [21]. Although lacking a CBM63, recombinantly produced loosenins from *Phanerochaete carnosa* (*Pca*LOOL) were shown to boost the enzymatic conversion of wood pulps and preparation of cellulose nanocrystals [22, 23], and increase the interfibril distances of wood-derived holocelluloses [24]. These studies on artificially modified substrates such as filter paper and wood pulps raised the question regarding the ability of microbial expansin-like proteins and loosenins to interact with cell wall polymers in native cell walls from hardwood trees. This study aims to evaluate the adsorption of expansin *Bs*EXLX1, loosenin *Pca*LOOL12, and a fusion of *Pca*LOOL12 with CBM63 from *Bs*EXLX1, to cell wall components of woody tissues from hybrid aspen using advanced microscopic techniques.

## Materials and methods

### Plant material

The fresh wood samples were collected from hybrid aspen (*Populus tremula L.* × *tremuloides* Michx. clone T89) trees grown for 9 weeks in the phenotyping platform (WIWAM Conveyor, custom designed by SMO, Eeklo, Belgium) [25]. To generate wood samples with partial removal of hemicelluloses, milled dry wood samples of hybrid aspen were sieved to 100–500 mm particle size and 1 g of sieved wood powder was subjected to subcritical water extraction (SWE) at 170 °C with buffered pH 5 for 60 min with an accelerated solvent extractor (ASE-300, Dionex, USA), according to method described elsewhere [26]. The non-extracted wood powder was used as control.

### Chemical composition analysis of wood powder

The aspen wood powder and the residue after subcritical water extraction were subjected to monosaccharide analysis by a two-step sulfuric acid hydrolysis [27]. In brief, 1 mg of sample was incubated with 125 μL of 72% H_2_SO_4_ at room temperature for 3 h, then diluted with 1375 μL of deionized water, and further incubated at 100 °C for 3 h. The hydrolysates were diluted 10 times with MilliQ water, filtered through a 0.2-mm syringe filter (Chromacol 17-SF-02-N) and analysed by high-performance anion exchange chromatography with pulsed amperometric detection (HPAEC-PAD) (ICS-6000 DC, Dionex) equipped with a CarboPac PA1 column (4 × 250 mm, Dionex) at 30 °C using the eluent gradients previously reported [28]. Monosaccharide quantification was performed by standard calibration (Ara, Rha, Fuc, Xyl, Man, Gal, Glc, GalA, MeGlcA and GlcA). The Klason lignin content was estimated from the gravimetric calculation after acid hydrolysis.

### Microbial expansin and loosenin cloning and production

The expansin-like protein from *Bacillus subtilis* (*Bs*EXLX1*,* GenBank accession no*.* WP_003231419.1*)* was recombinantly produced in *Escherichia coli* strain BL21 (DE3) from the codon-optimized gene subcloned to pET21a( +) plasmid. For protein expression, cells were incubated in LB medium at 37 °C until the optical density (OD_600_) reached 0.6, and 1 mM isopropyl β-D-1-thiogalactopyranoside (IPTG) was added for protein induction overnight. The cells were pelleted by centrifugation at 6000* g* for 20 min, the pellets were resuspended in lysis buffer (50 mM Tris–HCl, NaCl 200 mM, pH 7.8, Halt Protease Inhibitor, Invitrogen), and sonicated at 30% amplitude for 5 min. The cell debris was separated by ultracentrifugation at 33,000 g for 1 h, and the supernatants were mixed with His GraviTrap affinity column (GE Healthcare), washed with buffer supplemented with 20 mM imidazole, and eluted with an elution buffer (50 mM Tris–HCl, NaCl 200 mM, pH 7.8, 300 mM imidazole). The proteins were dialyzed to remove imidazole and sodium dodecyl sulphate and the protein purification was verified by polyacrylamide gel electrophoresis (SDS-PAGE). The final protein concentration was calculated by measuring the absorbance at 280 nm in a Nanodrop 2000C.

The loosenin-like protein from *Phanerochaete carnosa, Pca*LOOL12 (GenBank code EKM51974.1) [29] was recombinantly produced in *Komagataella phaffii* strain SMD1168H in accordance with the manufacturer’s instructions (Invitrogen, Thermo Fisher Scientific) and as previously described [18]. The *Pca*LOOL12-CBM63 fusion protein comprised a *Pca*LOOL12 at the N-terminus and CBM63 from *Bs*EXLX1 at the C-terminus of the protein. The two domains were connected by the linker sequence taken from *Bs*EXLX1, including two last amino acids from *Bs*EXLX1 domain 1 [18]. *Pca*LOOL12*-*CBM63 was recombinantly produced in *K. phaffii* as previously reported [18]. All the three recombinant proteins were purified with a His-tag on their C-terminal ends that served as a target for immunolabeling experiments.

### Expansin and loosenin treatment of woody biomass

The fresh wood samples from the 38th and 39th internodes were cut into blocks of 2 × 2 × 10 mm (radial, tangential and longitudinal) size. The proteins were diluted to 1 mg mL^−1^ using sodium acetate buffer (pH 5.0 for *Bs*EXLX1 and pH 6.0 for *Pca*LOOL12 and *Pca*LOOL12*-*CBM63). Between 5 and 6 wood blocks having a total weight of 6–7 mg or 6 mg of SWE residues and wood powder were transferred to 1.5 mL Eppendorf tubes containing microbial proteins loaded at 2% (w/w) and incubated at 25 °C for 48 h. Each treatment was carried out in triplicate.

### Microscopic analysis of treated biomass

After incubation with recombinant proteins, the samples were briefly washed in buffer and fixed in a mixture of 0.1% glutaraldehyde and 4% paraformaldehyde in 50 mM sodium cacodylate buffer for 4 h at room temperature and left overnight at 4 °C. After fixation, the SWE residue and wood powder samples were embedded in 4% agarose [30], and the solidified agarose was cut into 3 × 4 × 3 mm cubes. Both wood blocks and agarose cubes were then washed in phosphate buffer (pH 7.0), dehydrated in graded series of ethanol for 20 min from 30 to 95% (15 min each) to pure ethanol (× 3) and embedded in LR white resin as described elsewhere [31].

For bright field and immunofluorescence microscopy, 2-µm-thick transverse sections were prepared from LR white embedded blocks with a diamond knife. For general histological evaluation of the wood tissues, semithin sections were stained with toluidine blue O (0.5% aqueous). To confirm the protein interaction into deeper areas, these sections were taken after removing of 0.2-mm-thick surface layer from the blocks. Sections were mounted on formvar-coated slides and treated with 50 mM glycine/phosphate buffered saline (PBS) solution for 15 min. The sections were washed with PBS buffer and suspended in blocking buffer (PBS containing 3% skim milk) for 30 min at room temperature. Sections were incubated in anti-His-tag monoclonal antibodies (6x-His Tag monoclonal antibody, Alexa Flour 488, Thermo Scientific, USA, 1:100 dilution in PBS buffer) for 1 h at room temperature. After washing in PBS buffer, the sections were mounted in Fluoroshield (Sigma, Germany) on a clean glass slide and were examined using a Leica DMi8 inverted microscope (Leica Biosystems, Germany) fitted using 499 nm excitation and 520 nm emission. Immunofluorescence negative control sections were processed with omission of the antibody.

For immunogold labelling, ultrathin sections of 90 nm thickness obtained using ultramicrotome (Reichert Ultracut S, Leica, Austria) were mounted on copper grids, which were suspended in buffer A (PBS containing 1% bovine serum albumin and 0.1% NaN_3_, pH 8.2) for 30 min at room temperature. For the recombinant protein localization, the grids were incubated with anti-His-Tag antibody with 10 nm colloidal gold (10 nm Ni-NTA-Nanogold, Nanoprobes, USA) for 45 min, washed in three changes in buffer A and MilliQ water, and dried. For localization of glucuronoxylan and glucomannan, the grids were incubated with LM10 (for glucuronoxylan) and LM21 (glucomannan) monoclonal antibodies obtained from Plant Probes (UK) diluted 1:20 (V:V) in buffer A for 45 min. After three washings with buffer A for 5 min each, the grids were incubated with goat anti-rat secondary antibody labelled with 10-nm colloidal gold particles (BB International, UK) for 1 h at room temperature. For the negative immunolocalization control, the sections were processed with omission of primary antibodies. Finally, the grids were washed in six changes of buffer A for 15 min each, followed by washing with distilled water. All labelled sections were post-stained with 5% uranyl acetate for 30 min, washed in running distilled water for 1 min. All sections were examined under a transmission electron microscope (TEM, FEI TALOS L120C) at an accelerating voltage of 100 kV. The gold particle density quantification (per mm^2^ area) after LM10 and LM21 labelling was based on 20 measurements from five images per each triplicated sample. All measurements were taken using ImageJ software (National Institutes of Health, USA).

### Statistical analysis

Statistical analyses were performed in JMP Pro (v.16.0) software (SAS Institute Inc., Cary, NC, USA).

## Results

### Immunolocalization of *Bs*EXLX1,* Pca*LOOL12 and *Pca*LOOL12-CBM63 in the cell walls of wood fibres

**Fig. 1:**
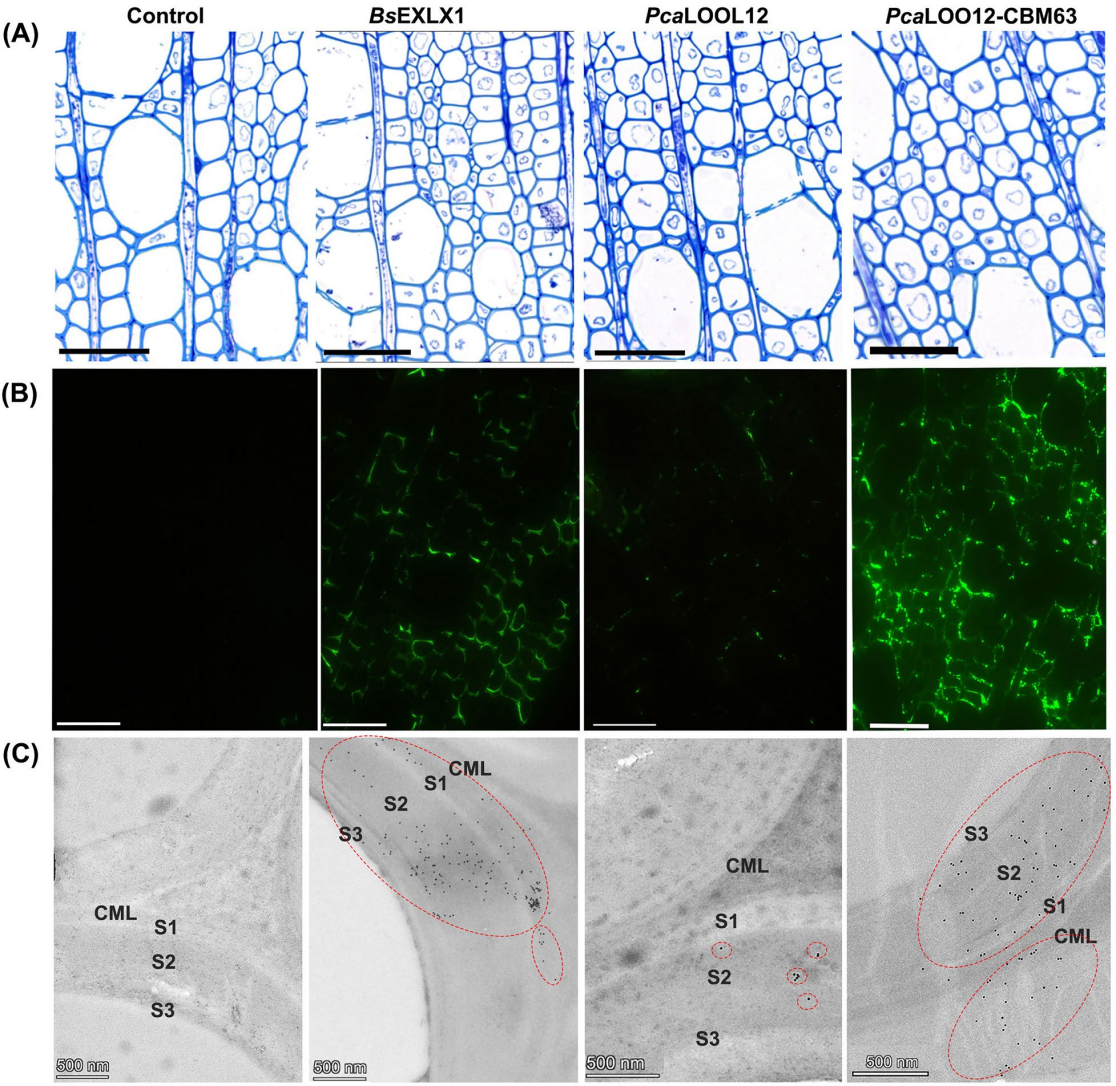
Light microscopy (**A**), immunofluorescence microscopy (**B**) and immunogold-transmission electron microscopy (**C**) images of transverse sections from fresh aspen wood samples treated with buffer (control), *Bs*EXLX1*, Pca*LOOL12 and *Pca*LOOL12-CBM63 proteins. Sections subjected to toluidine blue staining for general histology (**A**) and immunolabeling with anti-His antibody for recombinant protein localization within cell wall (**B**, **C**). Scale bar = 50 um (**A**, **B**), 500 nm (**C**). Compound middle lamellae (CML), secondary wall layers (S1, S2 and S3). Red dotted circles indicate areas of dense gold particle distribution

To evaluate the interaction of the microbial expansin-like (*Bs*EXLX1), loosenin (*Pca*LOOL12) and *Pca*LOOL12-CBM63 fusion proteins with lignified wood cell walls, the anatomy of developing wood was first examined using TBO-stained semithin wood sections (Fig. 1A). The selected wood areas containing secondary wall-thickened and lignified wood fibres were subjected to immunolabeling experiments. Strong fluorescence signals from the fibre cell walls of *Bs*EXLX1*-*treated samples in the selected wood zone area indicated the ability of the protein to interact with the lignified wood cell walls (Fig. 1B). For wood blocks treated with *Pca*LOOL12, a weak fluorescence signal was observed (Fig. 1B). By contrast, the samples treated with *Pca*LOOL12-CBM63 showed strong labelling. The differences in labelling among the three proteins were confirmed by transmission electron microscopy after immunogold labelling. A strong gold labelling for *Bs*EXLX1 and *Pca*LOOL12-CBM63 was evident in the secondary fibre wall, while a weaker immunogold labelling was observed in the *Pca*LOOL12 treated wood (Fig. 1C)*.* These results demonstrate the strong adsorption of *Bs*EXLX1 and *Pca*LOOL12-CBM63 fusion protein into the fibre secondary walls, since the signals were not washed out during immunolabeling procedure. The signals from both *Bs*EXLX1 and *Pca*LOOL12-CBM63 become more apparent in the secondary wall layers (S1, S2 and S3) compared to the compound middle lamellae (CML) (Fig. 1C). A reduced labelling in the CML could be due to a hindered binding of the proteins due to the higher lignin content and/or the different composition of CML versus secondary wall layers of hardwood fibres [32].

### Immunolocalization of hemicelluloses in the microbial protein-treated aspen wood fibres

**Fig. 2:**
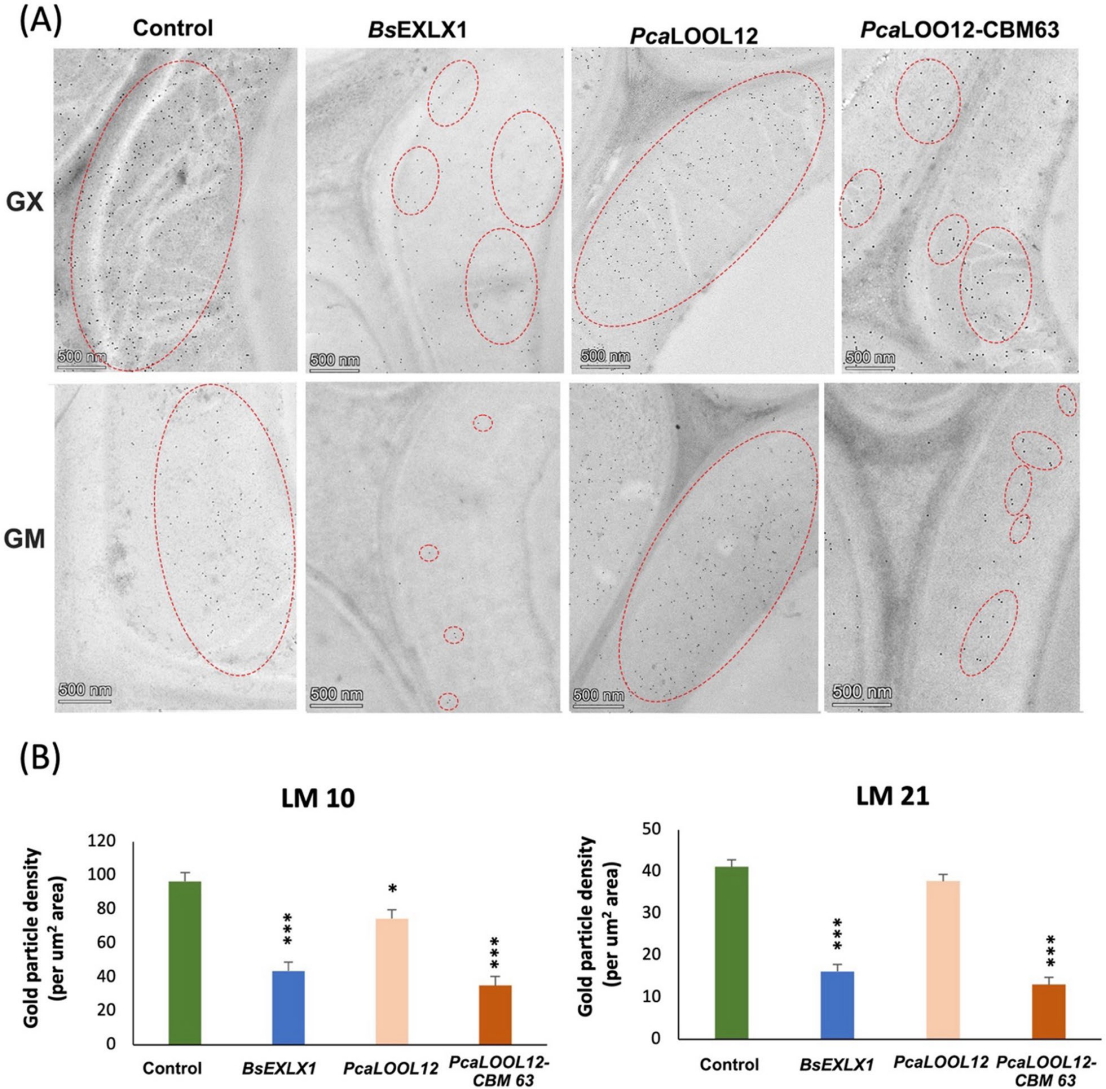
**A** Transmission electron microscopy (TEM) images from the transverse sections of control and protein-treated aspen wood, labelled with LM10 antibody for glucuronoxylan and LM21 antibody for glucomannan. **B** Density of gold particles (per mm^2^ area) in the control and protein-treated wood fibres using LM10 and LM21 labelling. *P ≤ 0.05; **P ≤ 0.01; ***P ≤ 0.001 for comparisons with control by Dunnett´s test. Red dotted circles indicate areas of dense gold particle distribution

The wood cell wall is characterized by spatial and temporal variation in the distribution of hemicelluloses and pectins. Therefore, to validate the possible relation between hemicellulose distribution and protein binding, we investigated the variation in the spatial distribution pattern of hemicelluloses in the secondary wall of aspen wood fibres. The localization of glucuronoxylan using LM10 antibody in a control tissue that was not incubated with microbial proteins revealed strong labelling in all the secondary wall layers (Fig. 2A), suggesting a homogenous distribution of glucuronoxylan in the secondary wall. A decrease in the gold particle distribution was observed in the S2 and S3 layers of *Bs*EXLX1 and *Pca*LOOL12-CBM63 treated samples (Fig. 2A, B).

The localization of glucomannan using LM21 antibody revealed relatively low labelling in S1 layer while S2 and S3 layer showed a higher density of gold particles, suggesting that glucomannans have certain spatial heterogeneity in the distribution pattern and/or in epitope exposure within the secondary wall layers of aspen wood fibres (Fig. 2A). The immunogold particle density measurements of LM21 labelling in control tissue (without protein treatment) was very low compared to LM10 labelling suggesting that glucuronoxylan is more abundant compared to glucomannan in the secondary wall of aspen wood fibres (Fig. 2A, B). Nevertheless, the LM21 labelling was clearly diminished following treatment with *Bs*EXLX1 and *Pca*LOOL12-CBM63 treated samples (Fig. 2F–H).

The decreased LM10 and LM21 signals from protein-treated samples compared to controls indicate that these proteins mask the glucuronoxylan and glucomannan epitopes. Our results suggest that the CBM63 facilitates binding between these proteins and hemicelluloses, and such interaction possibly restricts the glucuronoxylan and glucomannan recognition by LM10 and LM21 antibodies.

### Protein localization in wood fibres after partial removal of hemicelluloses

**Fig. 3:**
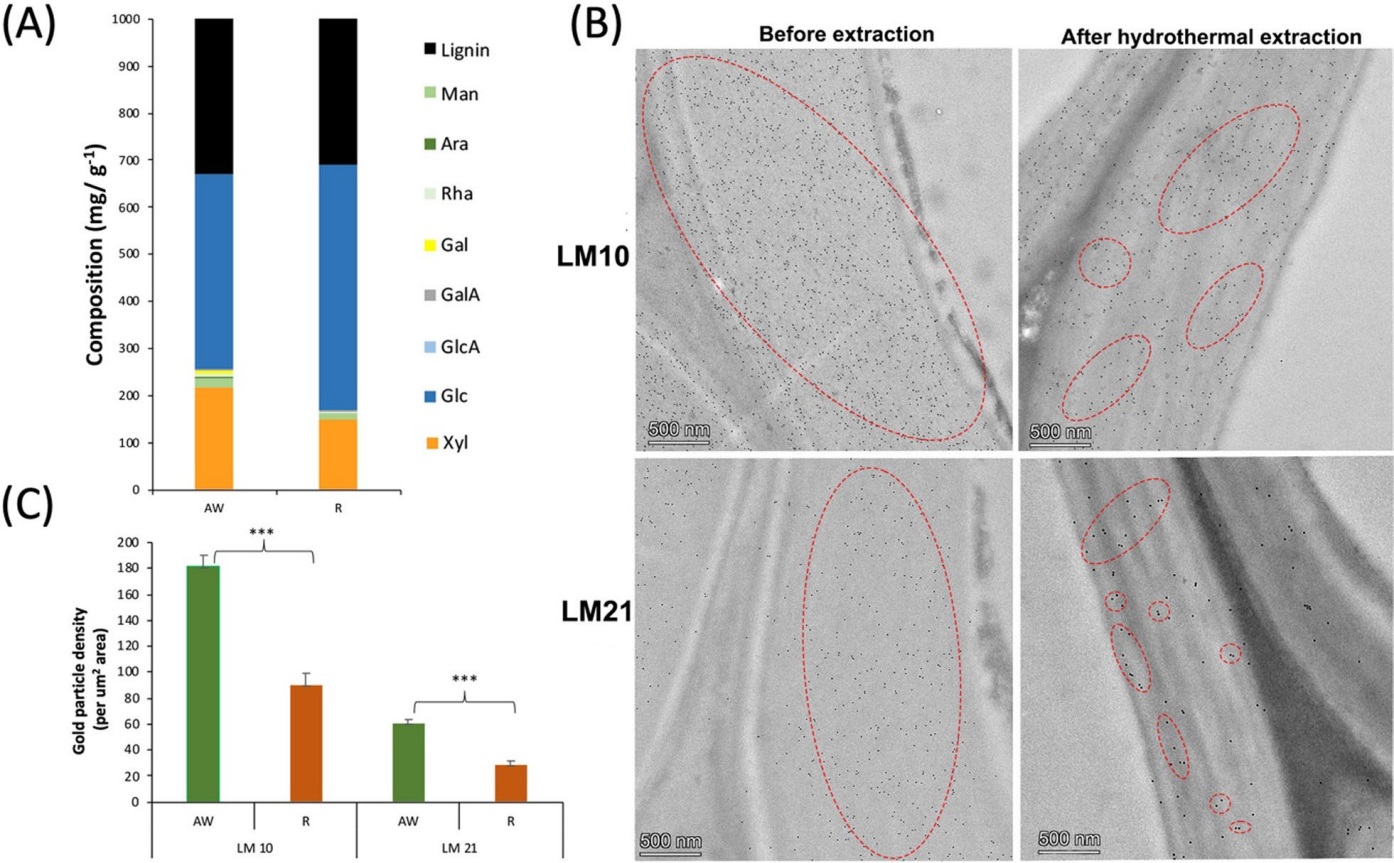
**A** Chemical composition of aspen wood (AW) and residue (R) of subcritical water extraction. **B** Transverse sections from fibres in the aspen wood and residue labelled for glucuronoxylan with LM10 antibody and glucomannan with LM21 antibody. **C** Density of gold particles (per mm^2^ area) for LM10 and LM21 labelling in the aspen wood and residue of subcritical water extraction. ***P ≤ 0.001 for comparisons with AW by Student’s *t*-test. Red dotted circles indicate areas of dense gold particle distribution. Red dotted circles indicate areas of dense gold particle distribution

The immunolabeling patterns described above suggest that the main interactions between the proteins and the hemicelluloses in aspen were mediated by the CBM63. To evaluate this hypothesis, we partially removed the hemicelluloses from the aspen wood powder using subcritical water extraction. SWE also removes a minor amount of extractable lignin, potentially in the form of lignin–carbohydrate complexes (LCCs), without largely affecting the cellulose and lignin content in the SWE residue [26, 33]. The chemical analysis of the SWE residue compared to the starting material revealed a 30–50% reduction in the monosaccharides attributed to glucomannan and glucuronoxylan (Fig. 3A). This decrease in glucuronoxylan and glucomannan was also confirmed by the decrease in immunolocalization signals and gold particle density of LM10 and LM21 in the SWE residue (Fig. 3B, C).

**Fig. 4:**
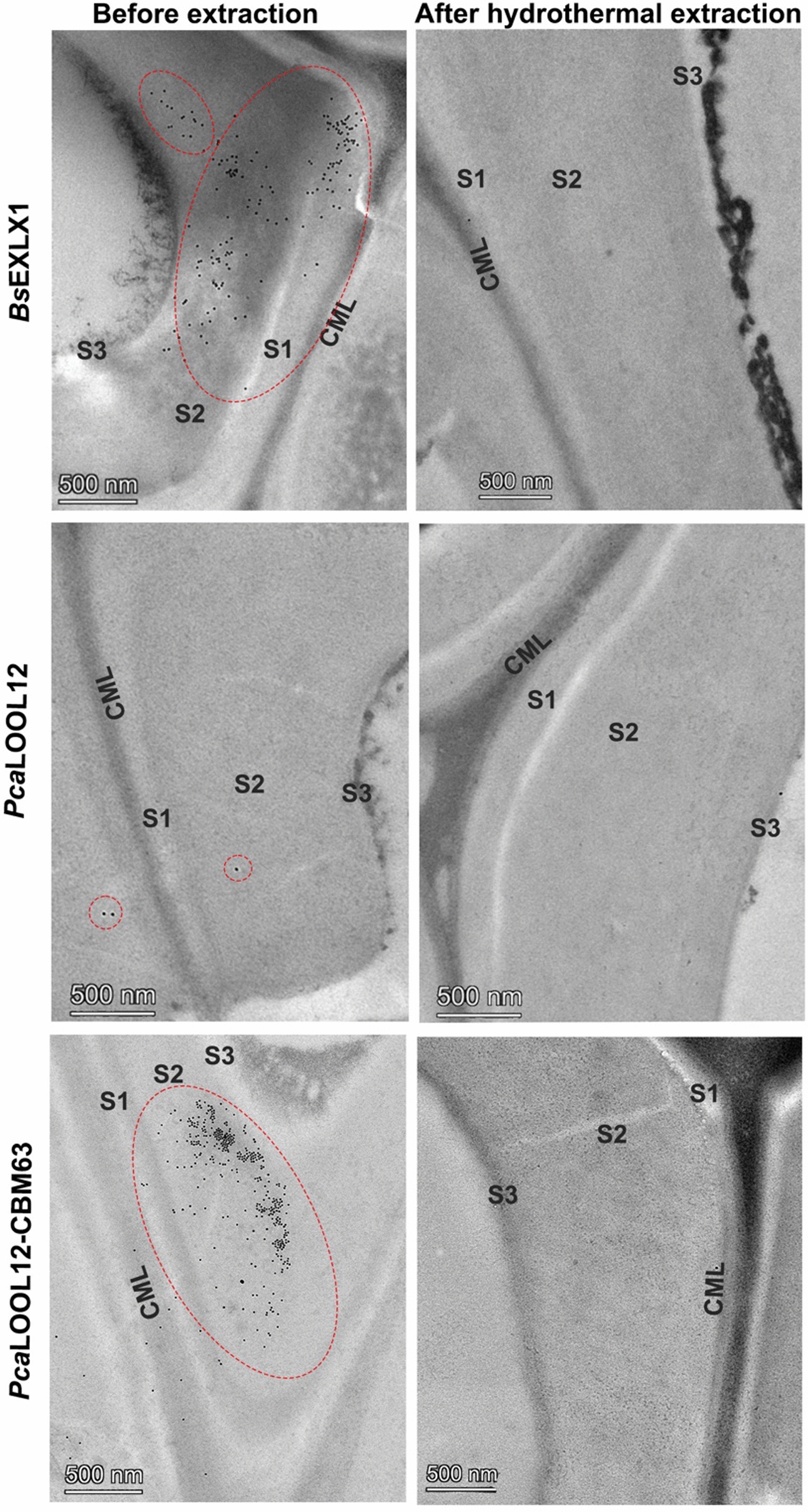
Ultrathin sections of agarose-embedded, recombinant protein (*Bs*EXLX1, *Pca*LOOL12 and *Pca*LOOL12-CBM63)-treated wood powder and SWE residue, immunogold labelled with anti-His tag antibody. Compound middle lamellae (CML), secondary wall layers 1 (S1), 2 (S2) and 3(S3). Red dotted circles indicate areas of dense gold particle distribution

To evaluate the effect of hemicellulose removal on the binding ability of the recombinant proteins, immunogold localization was performed in the protein-treated wood powder (starting material) and SWE residue entrapped into agarose for easy handling during processing and embedment in LR white resin. As observed for the fresh wood samples, the protein-treated wood powder (without SWE extraction) showed a strong labelling for *Bs*EXLX1 and the fusion protein *Pca*LOOL12-CBM63 but not for wild-type *Pca*LOOL12 (Fig. 4). After hemicellulose extraction, however, weak to no protein labelling was observed in the SWE residue (Fig. 4). These results support the hypothesis that hemicelluloses are needed for CBM63-mediated binding of *Bs*EXLX1 and the fusion protein *Pca*LOOL12-CBM63 to lignified secondary walls of aspen wood fibre.

## Discussion

So far, in situ studies of *Bs*EXLX1 and other expansin-related proteins have focused on primary cell wall tissues. On the other hand, secondary cell walls found in trees represent the most abundant carbon sink and source of renewable materials for biorefinery applications. In hardwoods, the secondary wall polysaccharide matrix is composed of glucuronoxylan and glucomannans; however, to our knowledge there have been no reports on the possible roles of these components in expansin-like protein binding. Our study now provides experimental evidence for the role of hardwood hemicelluloses (glucuronoxylan and glucomannan) as binding sites for CBM63 in *Bs*EXLX1 and the *Pca*LOOL12-CBM63 fusion protein in the secondary cell walls of aspen wood.

Consistent with earlier studies that demonstrate the importance of CBM63 to *Bs*EXLX1 binding to wheat coleoptile primary cell walls [21], the immunolocalization studies reported herein showed that appending the CBM63 to the C-terminus of *Pca*LOOL12 considerably increased loosenin binding to secondary cell walls of aspen wood. The immunolocalization studies also indicated a possible effect of the hemicellulose composition on the binding of CBM63-containing proteins to the aspen samples. For example, the density of immunogold-labelled *Bs*EXLX1 and *Pca*LOOL12-CBM63 was higher in secondary cell wall layers comprising glucuronoxylan and glucomannan compared to the CML region, which is characterized by comparatively high lignin and pectin contents as well as xyloglucan as the main hemicellulose [32]. Moreover, the labelling signal of LM10 and LM21 antibodies towards glucuronoxylan and glucomannan, respectively, decreased following pretreatment of the aspen samples with the CBM63-containing proteins, presumably through protein binding and masking the hemicellulose epitopes recognized by these antibodies. Consistent with this interpretation, partial hemicellulose removal by SWE substantially reduced the protein labelling signal observed in subsequently treated wood samples. Since SWE releases hemicelluloses from wood without substantially affecting lignin and cellulose contents [26, 33], this result also suggests that *Bs*EXLX1 and *Pca*LOOL12-CBM63 preferentially bind hemicelluloses over cellulose and lignin. Binding of *Bs*EXLX1 to hemicelluloses over other cell wall components has been observed using extracted wheat coleoptile primary cell walls [21], solid-state NMR analyses of *Arabidopsis thaliana* cell walls showing *Bs*EXLX1 binding to cellulose in close proximity to xyloglucan [34], as well as commercial sources of xylan, lignin and cellulose [35, 36]. The importance of glucuronoxylan and glucomannan to *Bs*EXLX1 and *Pca*LOOL12-CBM63 binding to the aspen wood samples now indicate these hemicelluloses could mediate expansin-related protein binding to the secondary cell walls of hardwood fibres.

Besides substrate selectivity, the depth of *Bs*EXLX1 and *Pca*LOOL12-CMB63 penetration into the aspen wood samples was investigated. Earlier immunolocalization studies of *Bs*EXLX1 in 0.5 mm-thick sections of celery petioles show strong surface binding of *Bs*EXLX1 to different cell types of xylem and phloem [37]. Herein, the wood sections were considerably thicker (2 µm) and strong immunolabeling signals from *Bs*EXLX1 and *Pca*LOOL12-CMB63 were observed 0.2 mm from the exposed sample surface. Whereas the tracheary elements in the primary xylem celery petioles are characterized by secondary walls in the form of spiral, annular and helical thickening in restricted cell wall areas [38], the tracheary elements in secondary xylem of higher woody plants have more complex pitted structure. Our study demonstrated that *Bs*EXLX1 and *Pca*LOOL12-CBM63 are able to bind within the structurally complex secondary wall of hardwood fibres.

In conclusion, immunolocalization methods were used herein to evaluate the interaction of microbial expansin-like protein *Bs*EXLX1 and loosenin *Pca*LOOL12, wild-type or fused with CBM63 (*Pca*LOOL12-CBM63), with wood fibres. We demonstrated the role of CBM63 in enhancing the binding of *Pca*LOOL12 to secondary cell wall components of aspen wood fibres. The masking effect of the proteins on glucuronoxylan and glucomannan labelling, and the weak immunolabeling of these proteins in wood fibres subjected to partial hemicellulose removal, suggested that CBM63-mediated binding occurs mainly through its interaction with hemicelluloses. Potential benefits of the protein binding to lignocellulose processing have not been tested but are worth investigating. In the case of enzymatic lignocellulose processing, careful consideration of protein loadings will likely be important to prevent potential competition of binding sites for expansin-related proteins and lytic enzymes.

## Data Availability

No datasets were generated or analysed during the current study.
